# Intra-crystalline protein diagenesis (IcPD) in *Patella vulgata*. Part II: Breakdown and temperature sensitivity

**DOI:** 10.1016/j.quageo.2012.08.001

**Published:** 2013-04

**Authors:** B. Demarchi, M.J. Collins, P.J. Tomiak, B.J. Davies, K.E.H. Penkman

**Affiliations:** a BioArCh, Departments of Biology, Archaeology and Chemistry, University of York, York YO10 5DD, UK; b School of Earth Sciences, University of Bristol, Wills Memorial Building, Queen's Rd, Bristol BS8 1RJ, UK; c Centre for Glaciology, Institute of Geography and Earth Sciences, Aberystwyth University, Llandinam Building, Penglais Campus, Aberystwyth SY23 3DB, UK

**Keywords:** *Patella vulgata*, Intra-crystalline proteins, High-temperature experiments, Kinetic parameters, Temperature sensitivity

## Abstract

Artificial diagenesis of the intra-crystalline proteins isolated from *Patella vulgata* was induced by isothermal heating at 140 °C, 110 °C and 80 °C. Protein breakdown was quantified for multiple amino acids, measuring the extent of peptide bond hydrolysis, amino acid racemisation and decomposition. The patterns of diagenesis are complex; therefore the kinetic parameters of the main reactions were estimated by two different methods: 1) a well-established approach based on fitting mathematical expressions to the experimental data, e.g. first-order rate equations for hydrolysis and power-transformed first-order rate equations for racemisation; and 2) an alternative model-free approach, which was developed by estimating a “scaling” factor for the independent variable (time) which produces the best alignment of the experimental data. This method allows the calculation of the relative reaction rates for the different temperatures of isothermal heating.

High-temperature data were compared with the extent of degradation detected in sub-fossil *Patella* specimens of known age, and we evaluated the ability of kinetic experiments to mimic diagenesis at burial temperature. The results highlighted a difference between patterns of degradation at low and high temperature and therefore we recommend caution for the extrapolation of protein breakdown rates to low burial temperatures for geochronological purposes when relying solely on kinetic data.

## Highlights

► High temperatures induce breakdown of closed-system proteins in *Patella vulgata*. ► Extent of racemisation, hydrolysis, decomposition quantified for multiple amino acids. ► Absence of leaching does not reduce the complexity of the diagenetic patterns. ► Kinetic parameters calculated by fits to 1st order models and a model-free approach. ► Relative rates of hydrolysis and racemisation are different at high/low temperature.

## 1 Introduction

The extent of protein diagenesis can provide a reliable estimate of the age since death of sub-fossil biominerals where the original protein fraction is preserved and has undergone *in situ* degradation (e.g. Brooks et al., 1990; Curry et al., 1991; Sykes et al., 1995; Penkman et al., 2008). Protein breakdown can be quantified in terms of peptide bond hydrolysis, amino acid racemisation (the interconversion reaction between l- and d- enantiomers of an amino acid) and amino acid decomposition (to either other amino acids or other organic compounds). However, the relationship between time elapsed since death of the organism and the extent of breakdown is complex and requires accurate evaluation of patterns of diagenesis as a function of both time and temperature. The lack of information about this relationship hampers the precision and accuracy of protein diagenesis as a numerical geochronological tool (e.g. Wehmiller, 1993).

High temperature experiments have traditionally been used to induce artificial protein diagenesis within laboratory timescales (e.g. Hare and Mitterer, 1969). The reliability of kinetic experiments for describing diagenesis in sub-fossil biominerals has been investigated in a number of studies (e.g. Wehmiller, 1980; Goodfriend and Meyer, 1991; Collins and Riley, 2000; Miller et al., 2000; Clarke and Murray-Wallace, 2006; Kaufman, 2006) which have highlighted some of the issues affecting the use of kinetic experiments to derive an adequate model of protein breakdown, and particularly amino acid racemisation:

(i) the use of mathematical expressions to describe racemisation generally underestimate the interplay of this with other diagenesis reactions (i.e. hydrolysis, decomposition);
(ii) the observation of outcomes (i.e. Arrhenius parameters for observed effects such as racemisation and hydrolysis) limits the reliability of high temperature experiments if the underlying concurrent reactions that contribute to the observed effect have different activation energies;
(iii) the loss of free amino acids (and soluble peptides) from an open system results in the under-prediction not only of rates of hydrolysis but also racemisation, in the latter case because the free amino acids are the most highly racemised.

Racemisation is thought to proceed via the base-catalysed abstraction of the α-proton in an amino acid and the formation of a carbanion intermediate (Neuberger, 1948). Consequently, highly alkaline environmental conditions may play an important role, but the effect of pH is likely to be less significant than that of temperature (see Orem and Kaufman, 2011). For free amino acids in aqueous solution, this reaction can be described by first-order reversible kinetics (FOK; Bada and Schroeder, 1975). However, in a series of high temperature investigations, the use of mathematical transformations on a range of biominerals (see a review in Clarke and Murray-Wallace, 2006) imply that these systems do not conform to simple kinetic models.

It has long been suggested that within a closed system, diagenesis should follow more predictable trajectories (Towe, 1980; Brooks et al., 1990; Sykes et al., 1995; Collins and Riley, 2000; Miller et al., 2000; Penkman et al., 2008). In avian eggshell (which shows closed-system behaviour, e.g. Brooks et al., 1990), isoleucine (Ile) epimerisation is hypothesised to obey (pseudo-) first order kinetics. The same pattern has been observed in both modern and fossil eggshells heated at different temperatures, and it was therefore concluded that Ile epimerisation was not hindered by an alternative rate-limiting step and that high-temperature kinetic experiments were able to accurately mimic diagenesis in the natural environment (Miller et al., 1999, 2000). Experiments on modern *Patella vulgata* have shown that the intra-crystalline proteins within this marine gastropod approximate a closed system from synthesis to analysis (Demarchi et al., 2013). Subsequently the problem of leaching (or diffusive loss) should be negligible, enabling diagenesis patterns to be investigated in terms of extent of hydrolysis, racemisation and decomposition (Wehmiller, 1980). We quantified the extent of breakdown induced by each reaction for multiple amino acids, allowing us to study a complex network of reactions occurring within the closed system and to compare the kinetic patterns displayed by different amino acids.

The aims of this paper therefore are:

1. to test the patterns of different diagenetic reactions displayed by intra-crystalline amino acids in *P. vulgata* at high temperatures;
2. to derive kinetic parameters for hydrolysis, racemisation and decomposition of multiple amino acids using alternative approaches;
3. to evaluate the potential for high temperature experiments to mimic diagenesis in the normal burial environment by comparing the breakdown patterns within heated and sub-fossil shells.

## 2 Materials and methods

### 2.1 *P. vulgata* specimens

Five modern live-collected shell specimens (collected in 2001 at St Mary's Lighthouse, near Newcastle, UK; experiments performed in 2007) were cleaned by rinsing and sonicating in ultrapure water (18.0 MΩ). One fragment from the shell apex and one from the shell rim were taken from each specimen and powdered in a quartz pestle and mortar (“bulk” sample). A second batch of shell powders (“rim only” sample) was prepared by selecting a fragment from the calcitic rim of each specimen and removing the aragonitic outer layer by drilling (see Demarchi et al., 2013). Both “bulk” and “rim only” batches of powdered shells included the medium and fine fractions only (i.e. 50–500 μm particle size).

Sub-fossil *P. vulgata* data used for comparison in this study come from UK sites of known age. The four Holocene sites are reported in Demarchi et al. (2011): Sand (Inner Sound, Western Ross, Scotland, radiocarbon dated to 7050–6450 cal BC) and Coire Sgamhadail 1 (Inner Sound, Western Ross, Scotland, radiocarbon dated to 2550–1880 cal BC) are detailed in Hardy and Wickham-Jones (2009); Archerfield (Dirleton, East Lothian, radiocarbon dated to 1410–1445 cal AD) and Whitegate Broch (Caithness, radiocarbon dated to 880–1210 cal AD). The Pleistocene raised beach deposit of Easington (North Yorkshire, UK) has been attributed to Marine Isotope Stage 7 (244-190 ka BP) in a comprehensive study by Davies et al. (2009). All the sub-fossil shells were sampled by selecting the calcitic rim only (Demarchi et al., 2013).

### 2.2 Bleaching step

In the light of the bleaching experiments described in Demarchi et al. (2013), all powdered shell samples were bleached (NaOCl, 12% w/v) for 48 h, as this pre-treatment ensures the removal of matrix proteins from *P. vulgata* and isolation of the intra-crystalline fraction.

### 2.3 Kinetic experiments

Kinetic experiments were performed by heating bleached *Patella* powders under hydrous conditions at 140 °C, 110 °C and 80 °C for various times (Table 1). The main experiment (heating at the three temperatures) was conducted on the “bulk” shell sample as this is more likely to give an average diagenetic pattern for the genus under investigation. However, as sampling the shell rim only was found to be more appropriate for sub-fossil *Patella* shells (Demarchi et al., 2013), a single set of kinetic experiments (140 °C) was performed on the “rim only” batch in order to provide comparative data.

The kinetic samples were prepared as follows (see also Demarchi et al., 2013): ∼20 mg of bleached powder was placed in sterile glass containers, 300 μL of ultrapure water was added and the sealed containers were placed in an oven for various times (Table 1). Three laboratory replicates were prepared for each time point. After heating, each replicate was split into four subsamples for the analysis of free amino acids (FAA) and total hydrolysable amino acids (THAA) from both the powder (p) and the supernatant water (w): THAAp, FAAp, THAAw and FAAw. Whilst a previous study described the results obtained from analysis of the amino acids recovered from both the water and the powder fractions (Demarchi et al., 2013), here we focus on the powder fraction only and therefore we use the acronyms THAA and FAA *in lieu* of THAAp and FAAp.

### 2.4 Chiral amino acid analysis

THAA samples were prepared by using a 24 h step of acid hydrolysis (20 μL 7 M HCl per mg of powder, at 110 °C); FAA samples were prepared by demineralising the powders in just enough cold 2 M HCl (a minimum of 10 μL 2 M HCl per mg of powder). After drying overnight in centrifugal evaporator, samples were rehydrated with the rehydration fluid routinely used in the NEaar laboratory, containing an internal spike of the non-protein amino acid l-homo-arginine. The extent of racemisation and hydrolysis for nine amino acids (aspartic acid/asparagine, Asx; glutamic acid/glutamine, Glx; serine, Ser; glycine, Gly; alanine, Ala; valine, Val; phenylalanine, Phe; leucine, Leu; isoleucine, Ile) was quantified by measuring the concentrations of d- and l- enantiomers by reverse phase high-performance liquid chromatography (RP-HPLC) following a modified method of Kaufman and Manley (1998) (see Penkman et al. (2008) for a more detailed description of the analytical method used in the NEaar laboratory).

## 3 Results and discussion

### 3.1 Hydrolysis

Hydrolysis progressively breaks the peptide bonds, releasing a complex mixture of products (Hill, 1965; Hare et al., 1975). This can occur by different processes, the most important being:

a) cleavage of an internal peptide bond, which is rapid for more hydrophilic amino acid residues (Bada, 1991);
b) internal aminolysis at the N-terminus, yielding diketopiperazines (Steinberg and Bada, 1983), which is more likely for small peptides made up of hydrophobic amino acids and at neutral pH;
c) hydrolysis of an amino acid at the C-terminus, which is acid/base catalysed but is independent of pH between pH 5–9 (Kahne and Still, 1988).

Hydrolysis has an observed effect on the racemisation rates (Hare, 1971; Hare et al., 1975; Wehmiller, 1980; Mitterer and Kriausakul, 1984). The widely accepted model (e.g. Riley and Collins, 1994) assumes that the progressive cleavage of the polypeptides will cause an increase in the number of N-termini (fast racemisation rates; e.g. Mitterer and Kriausakul, 1984). During the latter stages of diagenesis more modestly racemizing (free) amino acids become the dominant pool and the observed racemisation rates would be expected to decline (e.g. Kriausakul and Mitterer, 1980a, 1980b; Mitterer and Kriausakul, 1984). If hydrolysis occurs in a closed system, concentration data obtained from high temperature experiments should be able to clarify some of the reaction patterns, particularly for the latest stages of diagenesis (Wehmiller, 1980; Collins and Riley, 2000). If the system is open, loss of (the more highly racemised) FAA will have the effect of decreasing the observed rate and underestimating the rate of reaction.

#### 3.1.1 Extent of heating-induced hydrolysis

As some peptide bonds are more resistant to hydrolysis than others (Hill, 1965), and assuming no role for higher-order structures, there are 400 different rate constants (i.e. 20 × 20 amino acid pairs). For each amino acid the percentage of FAA is expressed as a fraction of the THAA measured on the same sample, for each time point:

(1) %FAA={[FAA]/[THAA]}×100

**Fig. 1:**
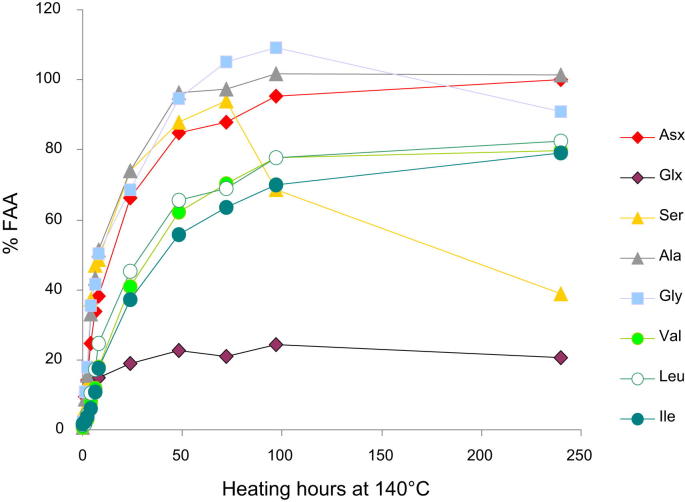
Percentage of free amino acids (% FAA) within bleached powders of *Patella* with progressive heating at 140 °C.

In *Patella*, % FAA in all amino acids analysed increases with increasing heating time, as hydrolysis proceeds (Fig. 1); for the three temperatures, the relative extent of overall release as free amino acids is:

Ala∼Ser∼Gly≥Asx≫Val(∼Leu∼Ile∼Phe)≫Glx

As expected, the peptide bonds of hydrophobic amino acids are less prone to hydrolysis, while more hydrophilic amino acids are released at a faster rate (Hill, 1965). However, this pattern can be complicated by the competing effect of amino acid decomposition. The simplest amino acids Gly and Ala show the most pronounced increase in % FAA, which is most likely due to the contribution of the decomposition of other amino acids, e.g. Ser, to the FAA pool (e.g. Bada et al., 1978). The slow increase observed for % FAA Glx over time is likely to be due to the difficulties in detecting FAA Glx, as this amino acid is preferentially released as a highly stable lactam and therefore unavailable for analysis in the FAA fraction (Vallentyne, 1964; Walton, 1998).

**Fig. 2:**
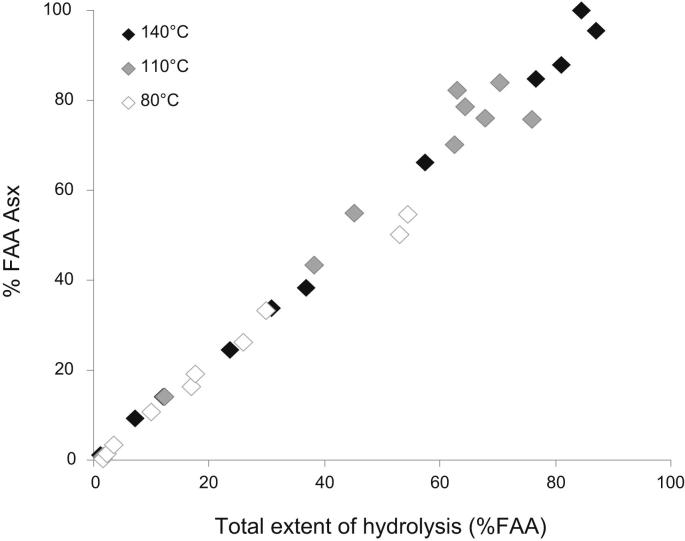
Increase of % FAA Asx within bleached *Patella* heated at 140 °C, 110 °C and 80 °C, normalised against the total % FAA at each time point; this was calculated as the sum of % FAA for Asx, Glx, Ser, Ala, Gly, Val, Phe, Leu and Ile.

The extent of hydrolysis for each amino acid, when compared to the overall extent of peptide bond breakdown (calculated as the ratio between the total [FAA] and the total [THAA] detected in the system) is also similar across temperatures (e.g. Asx in Fig. 2, a pattern found for all amino acids under consideration).

When considering the release of amino acids from the peptide chain, the range of values for % FAA should theoretically extend from 0% (no hydrolysis) to 100% (when the peptide chain is completely fragmented and only FAA are present). Complete hydrolysis of residues was only observed for Asx, Gly, Ala, L-Thr and Ser, and only during the 140 °C experiment (Fig. 1). Since a fraction of Ala and Gly is derived as a diagenetic product of other residues, it is important to note that the measurement of % FAA Ala and % FAA Gly does not necessarily reflect solely the hydrolysis of the original peptide-bound amino acids.

Most of the amino acids displayed a residual bound fraction, the relative abundance of which varies from amino acid to amino acid. At the highest levels of degradation seen (240 h heating at 140 °C), bound amino acids represent ∼20% of the initial concentration for Val, Ile and Leu, whilst almost 30% for Phe and ∼80% of the initial Glx (although the percentage of FAA Glx is underestimated due to lactam formation, see above).

A residual bound fraction, refractory to hydrolysis, has also been observed in the intra-crystalline proteins isolated from terrestrial gastropods (Penkman et al., 2008) as well as avian eggshells (Miller et al., 2000) and the whole-shell proteins from other biominerals (e.g. Hoering, 1980; Walton, 1998). If it is assumed that this fraction remains stable over long geological timescales, there are three alternative explanations for this observation (Collins and Riley, 2000). Firstly there is variation in the resistance of peptide bonds to hydrolysis, and the residual bound fraction may represent those residues most resistant to hydrolysis. Secondly, it may suggest a second order reaction, in which the hydrolysis of peptide bonds slows due to the increasingly limited availability of chemically available water within the closed system (Walton, 1998; Penkman et al., 2008). *Some* water should be initially present as fluid inclusions in the carbonate (Hudson, 1967; Towe, 1980; Gaffey, 1988); the amount of water has been found to be consistent within the same species but extremely variable between different species (between 0.6% and 2.2%) (Lécuyer and O’Neil, 1994). Water would also be generated as a chemical product of decomposition and condensation reactions (Bada et al., 1978; Collins et al., 1992). However, this pool of water may eventually run out, preventing further hydrolysis of the peptide bonds. Alternatively, the presence of the hydrolysis-resistant fraction might be explained by amino acids that are bound in hydrolysis-resistant compounds, e.g. the humic acids detected by Hoering (1980), despite the presence of water.

#### 3.1.2 Kinetic parameters: first-order rate equation

The rate of hydrolysis for the bleached *Patella* was estimated using the model of Miller et al. (1992) for pseudo-irreversible first order kinetics (pFOK)

(2) ln([Bound]/[Total])=−kt

where ln ([Bound]/[Total]) is the fraction of bound amino acids at a certain heating time (*t*, in seconds) and *k* is the apparent rate constant for hydrolysis at a specific temperature (s^−1^). This equation takes into account the effect of decomposition at any given heating time, although it is based on the assumption that only FAA can undergo decomposition.

Activation energies were calculated by estimating the reaction rates at 140 °C, 110 °C and 80 °C using Eq. (2) and extrapolating the kinetic parameters on an Arrhenius plot (Table 2). Glx parameters were not calculated due to the difficulties in detecting FAA Glx. For Ala and Ser, it was not possible to calculate the hydrolysis rates beyond “initial” diagenesis, due to the competing roles of decomposition and hydrolysis (see Section 3.3 below). Therefore only the “initial” hydrolysis activation energy was derived (reported in Table 2), but this can be compared with the “total” interval for other amino acids, because the upper limit considered here extended to ∼75% FAA Ala and 78% FAA Ser, similar to the % FAA Val and % FAA Ile considered for the maximum diagenetic point reached for those amino acids in these experiments.

**Fig. 3:**
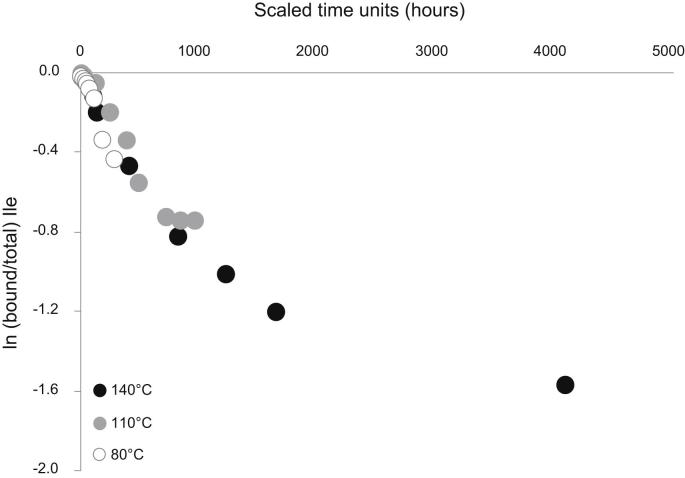
Extent of Ile hydrolysis in bleached *Patella* with progressive heating at 140 °C, 110 °C and 80 °C. The data were scaled to the heating hours at 110 °C on the x-axis in order to ease comparison across the three temperatures.

**Fig. 4:**
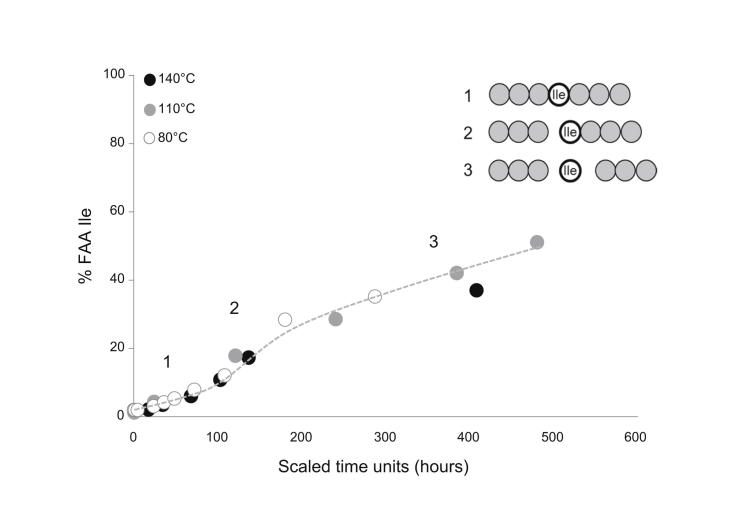
% FAA Ile for early diagenesis in bleached *Patella* upon isothermal heating at 140 °C, 110 °C and 80 °C. The data were scaled to the heating hours at 110 °C on the x-axis in order to ease comparison across the three temperatures. Note the initial lag in release of FAA Ile, interpreted as (1) difficult hydrolysis of peptide bonds involving Ile; (2) exposure of Ile at the chain termini and (3) release of FAA Ile.

If hydrolysis obeys pFOK, then the plot of ln ([Bound]/[Total]) versus time should yield a straight line with slope *k*. In *Patella*, that apparent release of FAA slows down progressively with increasing protein diagenesis (Fig. 3). A similar pattern has been observed in a range of biominerals that retain a closed system of proteins (e.g. Miller et al., 1992; Crisp et al., 2013; Tomiak et al., 2013). Due to the progressive slowing of the apparent rates of hydrolysis, a logarithmic model is not appropriate to mimic the patterns of “late” diagenesis (Fig. 3). Leaching of FAA would artificially dampen the apparent hydrolysis rate, so a linear relationship may not necessarily be expected for open systems. Within the closed system, as the intra-crystalline fraction of *P. vulgata* approximates (Demarchi et al., 2013), leaching cannot significantly affect the apparent hydrolysis rate. This implies that hydrolysis in *Patella* is not well approximated by a first-order rate equation and that the kinetic parameters derived based upon this assumption may be inaccurate. Indeed *Patella* does not conform to a pFOK model even during the earliest stages of the reaction, particularly for more hydrophobic amino acids, e.g. Ile (Fig. 4). We highlight the presence of a “lag” in the release of FAA, which we interpret (schematic in Fig. 4) to be produced by the difficult cleavage of peptide bonds involving hydrophobic amino acids (1) followed by limited chain scission which exposes the amino acid at the peptide termini (2) and, finally, release of terminal amino acids in the free pool (3).

#### 3.1.3 Kinetic parameters: a model-free approach

None of the data for hydrolysis or racemisation (see Section 3.2) conform to simple kinetic models (via first- or second-order reversible and non-reversible reactions) and therefore we argue that it is not ideal to derive rate estimates for *P. vulgata* from transformations based upon these models. We devised an alternative approach to the estimation of effective Arrhenius parameters, which estimates the relative rates of the reaction between different temperatures. This approach attempts to maximise the correspondence between different temperature experiments by scaling the time axis, thereby overcoming the complex patterns often formed in amino acid racemisation and decomposition kinetics. We observed that the patterns of hydrolysis, i.e. a plot of % FAA versus time, could be described by a third-order polynomial relationship and that this pattern is observed at the three temperatures used in the kinetic experiments. Therefore we did not attempt the linearisation of the data but we estimated a factor (“scaling factor”) which, if applied to the third-order polynomial, shifts the function on the time axis so that the data points at the three temperatures overlie. The data were normalised to the middle point, i.e. the 110 °C data series. The scaling factor is therefore synonymous with “relative rate”.

To capture the initial pattern of hydrolysis, the timescale was log-transformed, partly because the course of the experiments tends towards a logarithmic pattern and also because this reduces the bias on the end-point values, a limitation of power transformations. Log transformation means that a zero start time is not possible, therefore we fixed the initial value (which represents both decomposition/racemisation in the unheated samples prior to analysis and that induced during sample preparation) at log −1; this initial value was not included in the scaling algorithm for the abscissa.

The lower-temperature data (80 °C, 10 °C when available) describe more accurately the earliest stages of hydrolysis, whilst the density of the data points for the higher temperature data (140 °C) increases for the latest stages of the reaction (cf. plots in Supplementary Information 2). The polynomial functions often distorted away from the main trendline at both ends of the data range to encompass either extreme of the data set. Therefore the range over which the scaling of the abscissa was performed was selected from the ranges over which the ordinates for the two datasets under consideration overlap. Fitting was performed using a Generalized Reduced Gradient Algorithm (Microsoft Solver) to minimise the least squares difference between the two polynomial functions over the range selected for the two temperature pairs.

It is useful to calculate reaction rates over a similar diagenetic range (e.g. *D*/*L* values) across all the temperatures used in kinetic experiments (e.g. Kaufman, 2006). However, this limits the study to the earliest part of the diagenetic pathway. We propose that fitting the polynomial functions between pairs of temperatures over a range of values common to both overcomes this limitation, enabling a larger number of data-points to be considered, even if only in relationship to one other experiment. For completeness, we also estimated the scaling factors for hydrolysis by truncating the high-temperature data series at the same % FAA values (Table 3); when the nature of the data allowed this, the range for the 10 °C data was also truncated to exclude % FAA values lower than those observed in the high-temperature series (see Report Sheet, Supplementary Information 2).

We stress that fitting the functions to different ranges of values will result in different scaling factors being obtained, as shown in Table 3; because of the degree of subjectivity involved in the process of determining the optimal range over which the two polynomial functions are fitted, the ranges (and the least square differences) are reported alongside the relative rates of reaction (normalised to the middle time point, 110 °C; Table 3) and the Excel templates used for the calculations are included in the Supplementary Information 2.

The reaction rates, relative to the 110 °C data (*k*^rel^), were used to calculate the effective activation energies using a simple Arrhenius equation; *E*_*a*_ values reported in Table 3 were obtained by using the high-temperature data only, whilst values obtained when including the available 10 °C data are included in Supplementary Information 2 (Report Sheet). We note that when the fitting is performed over truncated data series, the resulting effective *E*_*a*_ values are not heavily affected (maximum difference = 2 kJ/mol for Ile) except for Gly and Ser; however, for these amino acids the detected concentration of FAA is affected by decomposition and the fitting of the third-order polynomial to the data series was generally not very good.

The pre-exponential factors (A) obtained are comparable across the different amino acids, but the absolute values depend on the arbitrary choice of normalising the data to the 110 °C experiment (*k*^rel^ 110 °C = 1); therefore these values are not reported in Table 3 but can be found in Supplementary Information 2.

#### 3.1.4 Temperature sensitivity of hydrolysis in *Patella*

The activation energy of hydrolysis when estimated using pFOK model could only be calculated for six of the amino acids, due to the poor fit to the experimental data (Table 2). The range of activation energies for these six amino acids is 84–108 kJ/mol; however, for three of these (Val, Leu, Ile), the pattern fell so far from the expected model that a second rate estimate was calculated based upon the earliest phase of diagenesis. When these rates are considered, the activation energies are much higher (98–132 kJ/mol).

Collins and Riley (2000) compiled activation energy values from a variety of studies on peptides and proteins, finding a range of 83–99 kJ/mol (Hare, 1976; Kriausakul and Mitterer, 1980b; Qian et al., 1993). The values obtained for “complete diagenesis” in this study using pFOK fall within this range, except for Asx. The values for the “early diagenesis” of Val (118 kJ/mol) and Ile (132 kJ/mol) were higher than those for Ser (97 kJ/mol) and Asx (108 kJ/mol), and higher than the value for the “early diagenesis” of Leu (115 kJ/mol), which was close to that obtained by Miller et al. (1992; 114 kJ/mol, ostrich eggshell).

Conversely for the model-free approach, the rates for nine amino acids could be estimated and all but Ser were found to range from 111 to 128 kJ/mol (Table 3). Due to the competing effect of hydrolysis and decomposition, Ser yielded the worst fit of the experimental data to the third-order polynomial, and significantly lower activation energy than the other amino acids (87 kJ/mol).

### 3.2 Racemisation

#### 3.2.1 Extent of THAA racemisation

**Fig. 5:**
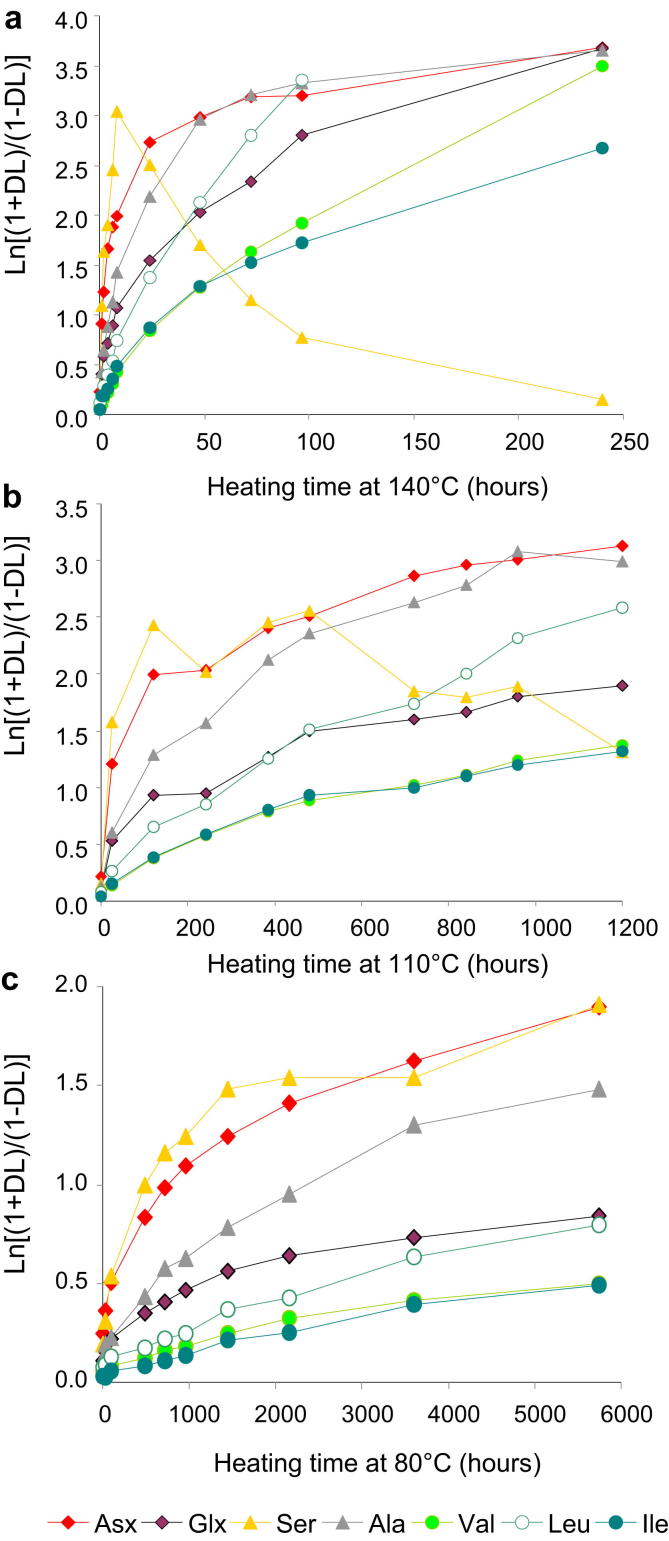
Extent of racemisation expressed as the first-order reversible rate law Ln[(1 + *D*/*L*)/(1 − K′*D*/*L*)] for THAA Asx, Glx, Ser, Ala, Val, Leu and Ile at 140 °C (a), 110 °C (b) and 80 °C (c).

Racemisation within the closed system in *Patella* does not follow first-order reversible kinetics (Fig. 5), i.e. the plot of ln[(1 + *DL*)/(1 − *DL*)] against heating time does not produce a straight line for any of the well-resolved amino acids. This is due to the network of reactions occurring within the system, which affect the position occupied by each amino acid within the protein sequence (i.e. interior, terminal; see Mitterer and Kriausakul, 1984), and therefore their ability to racemise.

The racemisation patterns in *Patella* are complex during isothermal heating. The extent of amino acid racemisation varies as diagenesis proceeds in time and at different temperatures (Fig. 5a, b and c). At 80 °C, the relative order over the timecourse covered within this study is:

Ser=Asx≥Ala>Glx=Leu≥Val∼Ile(Fig.5c)

Similarly, between 0 and 480 h at 110 °C and 0–24 h at 140 °C, we found that:

Ser≥Asx>Ala≥Glx≥Leu>Ile>Val(Fig.5aandb)

These relative apparent racemisation rates are similar to those reported by Smith and Evans (1980) for FAA in aqueous solution. Higher racemisation rates of Ser compared to Asx have been observed at pH 7.6 and explained in terms of an enhanced electron-withdrawing effect of the β-OH group of serine in comparison with the ionised β-COOH of aspartic acid (Steinberg et al., 1984). However, after 24 h of heating at 140 °C and 480 h of heating at 110 °C, Ser displays a clear reversal in *D*/*L* values, presumably due to its rapid decomposition to racemic Ala. This reversal has also been observed in closed system proteins isolated from terrestrial gastropods and other biominerals, e.g. Penkman et al. (2008), but also Kaufman (2006). The rates of Asx and Ala are similar (possibly due to the contribution of Ser decomposition to the overall extent of Ala racemisation) (see Section 3.3 and Supplementary Information 3), while Leu racemises at a faster rate than Glx, contrasting with what is observed during the first interval of diagenesis (Fig. 5a and b).

#### 3.2.2 Extent of FAA racemisation

**Fig. 6:**
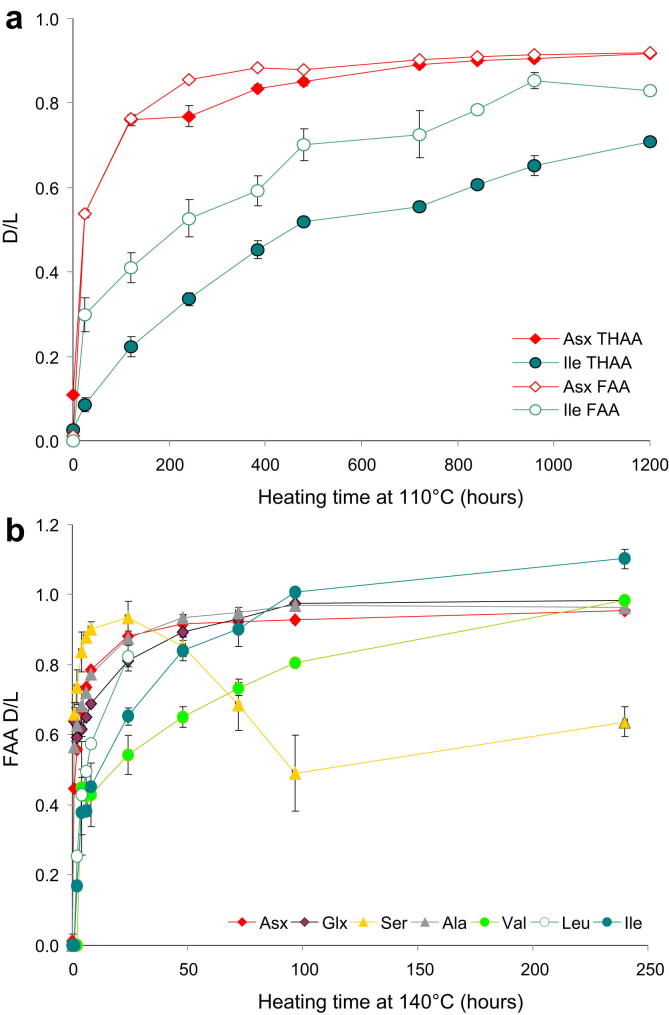
(a) Extent of THAA and FAA racemisation (*D*/*L* values) for Asx and Ile in bleached *Patella* upon heating at 110 °C; (b) Extent of FAA racemisation for Asx, Glx, Ser, Ala, Val, Leu, Ile in bleached *Patella* heated at 140 °C for various times.

Free amino acids are released in the closed system mainly by hydrolysis of the peptide bonds. The extent of FAA racemisation of all amino acids is higher than for their THAA counterpart (Fig. 6 and Supplementary Information 4); this fits with the model that hydrolysis releases highly racemised terminal amino acids in the system, although the rates of FAA racemisation may be lower (Mitterer and Kriausakul, 1984).

An exception is the in-chain racemisation of Asx via a succinimidyl intermediate (Geiger and Clarke, 1987; Stephenson and Clarke, 1989). Here we observed similar extents of racemisation for FAA and THAA Asx (Fig. 6a). This suggests that the mechanism that releases racemised FAA Asx is different from that operating for the other amino acids. The value for starting level of the extent of THAA Asx racemisation is at ∼0.1; this is likely to reflect both preparation-induced racemisation and the extent of Asx degradation due to the natural ageing of the six-year old *Patella* specimens.

The relative order of FAA racemisation for multiple amino acids in *Patella* is similar to that observed for the THAA fraction (Fig. 6b). Ser displays a reversal after 480 h heating at 110 °C and after 24 h at 140 °C, where Ser *D*/*L* ∼0.95, but not during the 80 °C experiment, because *D*/*L* values only reach ∼0.95 by the longest time point at 80 °C. FAA *D*/*L* values could not be determined for unheated samples, due to the low concentration of FAA amino acids (<15 pmol/mg), and were therefore assumed to be equal to 0. Similarly, FAA *D*/*L*s for Val, Ile and Leu could not be determined accurately for the first few time points: up to 2 h at 140 °C, 24 h at 110 °C and 96 h at 80 °C.

#### 3.2.3 Kinetic parameters (THAA): constrained power-law kinetic (CPK)

It is well known that amino acid racemisation in biominerals does not conform to FOK and a variety of mathematical approaches have been used in the past to overcome this (Clarke and Murray-Wallace, 2006). In order to derive the kinetic parameters for racemisation of different amino acids, we applied a power transformation of the integrated rate equation for first-order reversible kinetics, or constrained power-law kinetic (CPK) transformation, following Manley et al. (2000):

(3) $$\left(1+{\text{K}}^{\prime }\;\right)kt+\text{C}={\left[\left(1+D/L\right)/\left(1-\left({\text{K}}^{\prime }D/L\right)\right)\right]}^{n}$$

where *k* is the forward rate constant, K′ the reciprocal of the equilibrium constant K (K′ = 1 for most amino acids except Ile, where K = 1.3), *t* is the heating time (in seconds), C is a constant, determined experimentally, which represents the right-hand side term of the equation at *t* = 0, *n* is the exponent which yields the best fit of the experimental data to a linear relationship with time.

**Fig. 7:**
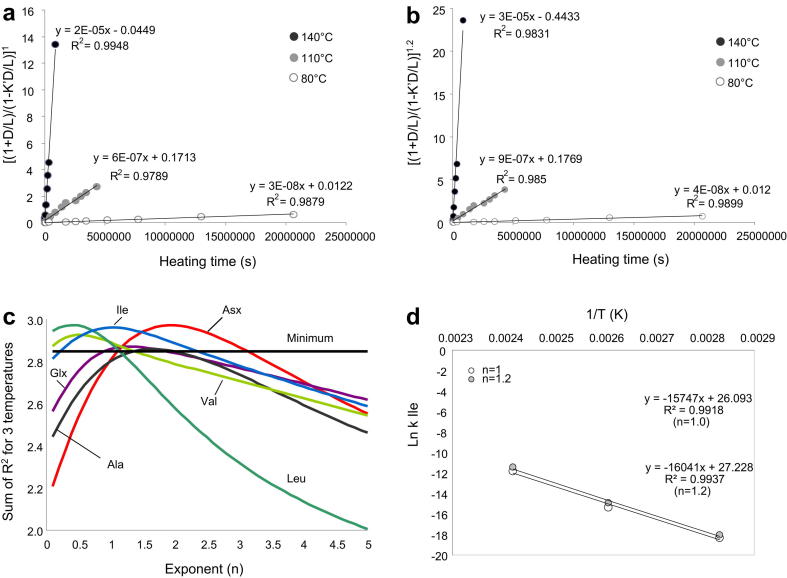
(a) Ile epimerisation rates at 140 °C, 110 °C and 80 °C estimated by raising the integrated first-order rate equation to the exponent *n* = 1.2, which yields good linearization of the experimental data for most of the amino acids. (b) Ile epimerisation rates at 140 °C, 110 °C and 80 °C estimated by raising the integrated first-order rate equation to the exponent that yielded the best fit to the experimental data (*n* = 1). (c) Evaluation of the “best fit” exponent to be used to linearise the experimental data at 140 °C, 110 °C and 80 °C for multiple amino acids; the maximum of each curve represents the highest value of the sum of the *R*^2^ for the correlation between the modified rate equation and the experimental data and indicates the best value of the exponent *n* to be used in Eq. (3). (d) Arrhenius plot for Ile epimerisation.

The trajectory of increase in the extent of racemisation in time at different temperatures can be approximated by a power function (Kaufman, 2000, 2006; Manley et al., 2000), with different exponents yielding the best fit for different amino acids (Fig. 7). Raising the [(1 + *D*/*L*)/(1 − K′*D*/*L*)] term to the same exponent of 1.2 yields adequate correlation coefficients for all amino acids except Val and Leu, which are best approximated by a square root function (i.e. a power function of 0.5). In order to evaluate the goodness of the fit to the data in a simple way, we defined a minimum value for the sum of the *R*^2^ for the three temperatures: i.e. if *R*^2^ ≥ 0.95, then the sum should be ≥2.85. However, for comparative purposes, we also estimated the apparent reaction rates by raising the right-hand side term of Eq. (3) to the exponent which yielded the best fit to the data, i.e. the maximum of each curve in Fig. 7.

The kinetic parameters and the coefficient of determination (*R*^2^) obtained for the seven amino acids considered here are reported in Table 4. The ranges for the activation energy values are 110–150 kJ/mol and 121–149 kJ/mol for exponent 1.2 and for “best fit” exponent, respectively. Asx displays the lowest activation energy (∼110 kJ/mol) when an exponent *n* = 1.2 is used; however, the best fit of the data is given by *n* = 1.9, which yields an activation energy of 131 kJ/mol. Other significant differences between the activation energies obtained with different values of *n* were found for Ser, Ala, Val and Leu. Conversely, the two *n* values (“best fit” and *n* = 1.2) for Glx and Ile were similar and this is reflected by the comparable *E*_*a*_ values we calculated.

The activation energies for racemisation have been calculated by applying similar constrained power-law kinetic transformation in a range of biominerals: Kaufman (2000) obtained *E*_*a*_ = 123.4 kJ/mol for both Asx and Glx in the ostracode *Candona* and 131 and 132 kJ/mol respectively for the foraminifera *Pullienatina* (Kaufman, 2006); Manley et al. (2000) found Asx *E*_*a*_ = 125.9 kJ/mol for the mollusc *Mya* and 126.2 kJ/mol for *Hiatella*, values that compare well with those estimated by Goodfriend et al. (1996) for Asx on the same molluscan genera (∼126 and ∼128 kJ/mol, respectively). In this study we estimated a large range of *E*_*a*_ values using power transformations, which can be either much lower (Asx, when *n* = 1.2) or much higher (Glx, when *n* = 1.2 and *n* = 1.3; Ser, when *n* = 2.8; Ala, when *n* = 1.7; Val, when *n* = 1.2; Leu, when *n* = 1.2) than the values reported for other biominerals.

#### 3.2.4 Kinetic parameters (THAA): a model-free approach

A similar approach to that used to estimate the relative rates of hydrolysis was also applied to the calculation of the *effective* Arrhenius parameters for racemisation. We estimated the “scaling” factors that produce the best alignment of the data across the three temperatures (see Section 3.1.3) by fitting a third-order polynomial to the raw *D*/*L* data and used the relative rates thus obtained to calculate the effective kinetic parameters (Table 5 and Supplementary Information 1). Table 5 reports the values obtained when two pairs of data series (i.e. 80 °C and 110 °C, 140 °C and 110 °C) are fitted over the whole range of *D*/*L* values and those obtained by truncating the data series for an interval of *D*/*L*s which is common to the three temperatures of the kinetic experiments. The effect of truncating the data series on the scaling factors is more pronounced for racemisation than for hydrolysis (see Section 3.1.3), particularly for Asx, Glx, Ser, Ala, Val and Leu.

The range of *E*_*a*_ values by fitting pairs of data series over the whole range varies between 128 and 145 kJ/mol, with Glx displaying the highest temperature sensitivity; if the values obtained by scaling the data series over a limited (truncated) range of *D*/*L*s are considered, the overall range is reduced (126–141 kJ/mol). The discussion of the temperature sensitivities of racemisation and hydrolysis (below and in Section 3.4) is based upon the values obtained by fitting the polynomial functions between pairs of temperatures and including a range of values which is common between the two, as we propose that this provides a more complete picture of diagenesis.

The variability of the effective *E_a_* values obtained by the scaling approach is comparable to that obtained by using a transformed first-order rate equation. The relative order obtained by using the two approaches is also similar: Glx displays the highest temperature sensitivity (except for Ser when estimated by CPK), Leu and Val the lowest. However, the absolute values of the activation energies for Asx, Glx, Leu, Ile and Val estimated with the model-free approach are higher (Tables 4 and 5). Conversely, E_a_ for Ala and Ser is lower when estimated by the model-free approach (128 kJ/mol versus 140 kJ/mol for Ala, 133 kJ/mol versus 149 kJ/mol for Ser).

The slightly higher variability of the E_a_ values obtained for racemisation compared to that for hydrolysis (σ_racemisation_= 6 kJ/mol versus σ_hydrolysis_=5 kJ/mol, excluding E_a_ hydrolysis of Ser) probably reflects the range of mechanisms involved in the observed racemisation of different amino acids within biominerals, whilst in contrast peptide bond hydrolysis is likely to occur according to the same mechanism (albeit the *rates* of hydrolysis are different for different amino acid pairs).

### 3.3 Decomposition

Within a closed system, where leaching can be considered negligible, the quantification of the amino acid concentration allows reliable calculations of the kinetic parameters for amino acid decomposition, the third most important diagenetic pathway followed by amino acids (Wehmiller, 1980).

**Fig. 8:**
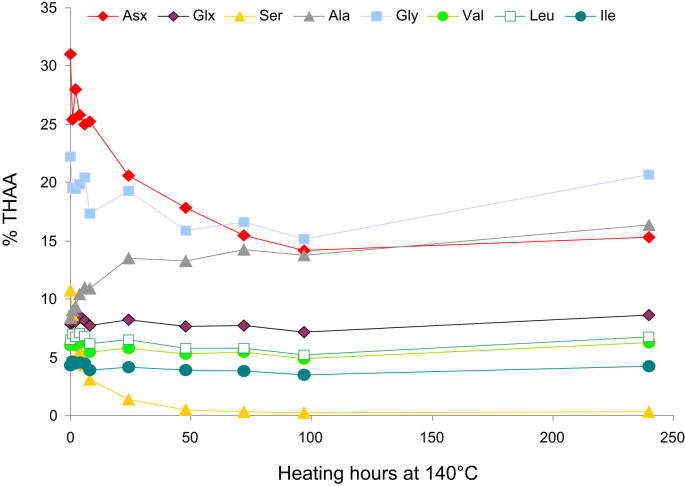
Variation of [THAA] Asx, Glx, Ser, Ala, Gly, Val, Leu, Ile during isothermal heating at 140 °C, expressed as the percentage of [THAA] of each amino acid relative to the sum of the [THAA] of the same amino acids within unheated, bleached *Patella* shells.

Within the intra-crystalline fraction isolated from *Patella* we observed a rapid decrease of the THAA concentration of Ser, L-Thr and Asx, whilst Leu, Ile, Val and Glx decreased very slowly; in contrast [Ala] and [Gly] increased. Fig. 8 shows the initial percentage of each amino acid (in unheated samples) and their change with heating time, at 140 °C; the same pattern is repeated at all three temperatures, and has also been highlighted for protein degradation within avian eggshells (Miller et al., 2000).

Decomposition pathways are complex and little information is available on their kinetics. Asp undergoes reversible decomposition to ammonia and fumaric acid or irreversible decarboxylation of the α- or β-carbons to form Ala or β-Ala (Bada and Miller, 1970; Walton, 1998). Moreover, ammonia is also released by Asn (and Gln) deamination to Asp (and Glu). The amount of ammonia released is difficult to quantify under the experimental and analytical conditions used here, therefore we did not attempt to derive the kinetic parameters for a specific decomposition pathway of Asx.

Dehydration, aldol cleavage and decarboxylation are the three main pathways for Ser and Thr decomposition. Ser dehydration yields Ala, aldol cleavage yields Gly and formaldehyde, whilst ethanolamine is formed by decarboxylation (Vallentyne, 1964; Bada et al., 1978; Walton, 1998). In the free state, Ser dehydration to Ala is the prevalent reaction (Bada and Man, 1980). In this study, we observed an increase for both Gly and Ala concentrations. This means that both aldol cleavage and dehydration of Ser may be occurring; however, Gly is formed by the decomposition of both Thr and Ser, which complicates estimates of the decomposition rates. For simplicity, here we assume that Ser dehydration is the main reaction for purposes of calculation of the kinetic parameters of the reaction; this is also supported by the observations of Bada et al. (1978) and Walton (1998).

For both Ser and Asx decomposition, we applied a logarithmic model (Bada et al., 1978) approach for the estimate of the Arrhenius parameters:

(4) $$\text{ln}\left({\left[\text{THAA}\right]}_{\text{aa}}/{\left[\text{Total}\;\text{THAA}\right]}_{\text{0}}\right)=-kt$$

where [THAA]_aa_ is the THAA concentration of a specific amino acid and [Total THAA]_0_ is the total THAA concentration for all amino acids in the system, at time *t* = 0 (i.e. in unheated samples). Although values of *R*^2^ < 0.9 were found for the regression of Eq. (4) for the lowest temperature, we were able to roughly estimate the kinetic parameters for Ser and Asx decomposition, obtaining values of *E*_*a*_ Asx = 109 kJ/mol and *E*_*a*_ Ser = 103 kJ/mol (Table 6).

The model-free approach was also used to determine relative decomposition rates of Asx and Ser at high temperature (80–140 °C); the [THAA]_aa_/[Total THAA]_0_ factor (defined above) was plotted against the “scaled” logarithm of time and the effective Arrhenius parameters were estimated (Table 7). The values derived with this approach are higher (*E*_*a*_ Asx ∼146 kJ/mol, *E*_*a*_ Ser ∼ 131 kJ/mol) than those obtained by using Eq. (4). The effective activation energy of decomposition of Ser to Ala was estimated by fitting a third-order polynomial to the raw [Ser]/[Ala] THAA data; the value thus obtained (131 kJ/mol) is the same as that obtained when considering [THAA]_Ser_/[Total THAA]_0_ in *Patella*, but slightly higher than that obtained by Vallentyne (1964) for decomposition of Ser in aqueous solution (123 kJ/mol).

### 3.4 Temperature sensitivity of protein diagenesis in *P. vulgata*

The observed extent of diagenesis in biominerals is the result of a complex network of reactions, each characterised by its specific temperature sensitivity. We have estimated the kinetic parameters for racemisation, hydrolysis and decomposition for multiple amino acids; however, these are likely to be affected by significant errors. Uncertainties derive from multiple sources, including: a) the analytical precision of the technique; b) the variability of the three laboratory replicates for each time point; c) potential variation of the oven temperatures during isothermal heating of the samples; d) the fitting of different curves through the experimental data for the estimates of the reaction rates; e) the fitting of a straight line through the calculated reaction rates on an Arrhenius plot. For example, McCoy (1987) estimated an uncertainty around 2% for the activation energy of isoleucine epimerisation in *Lymnaea* shells heated at high temperatures.

Nonetheless, the range of values obtained for the three reactions within the intra-crystalline protein fraction within *Patella* are different enough to be able to draw some conclusions on the role played by each reaction on the overall extent of diagenesis.

**Fig. 9:**
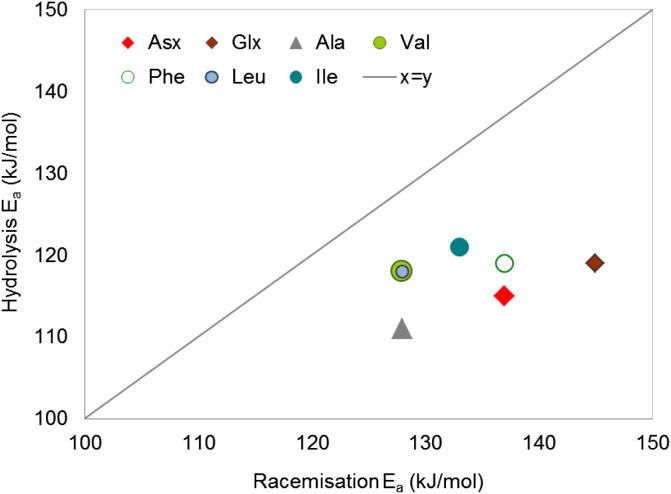
Effective activation energies (*E*_*a*_, kJ/mol) obtained with the “scaling” method for racemisation and hydrolysis for multiple amino acids; note that *E*_*a*_ racemisation >*E*_*a*_ hydrolysis.

The activation energies for hydrolysis are generally lower than for racemisation for all amino acids considered here (Asx, Glx, Ser, Ala, Val, Phe, Leu, Ile) and irrespective of the mathematical approach used to estimate the reaction rates. The only exceptions are Asx and Ile (early diagenesis only, Table 4) when a pFOK rate equation is used for both hydrolysis and racemisation: the *E*_*a*_ values for the two reactions are similar. In all other cases, and always for values estimated with our “model-free” approach, hydrolysis appears to be less temperature sensitive than racemisation (Fig. 9). The offsets vary according to the amino acid and the method used to estimate the Arrhenius parameters; Ser displays the largest difference between racemisation and hydrolysis (46 kJ/mol) but due to the competing effect of decomposition (*E*_*a*_ = 131 kJ/mol) the overall effective temperature sensitivity for Ser degradation it is difficult to evaluate. If Ser is excluded, the average offset between hydrolysis and racemisation for the other amino acids is 15 ± 7 kJ/mol.

#### 3.4.1 Patterns of diagenesis at high and low temperature

The offset between temperature sensitivities of racemisation and hydrolysis may have an impact on the overall patterns of diagenesis observed at high temperature and at the normal burial temperature at which sub-fossil samples are typically exposed. Patterns of hydrolysis and racemisation within a closed system can be examined as they proceed by plotting the % FAA of a given amino acid against the *D*/*L* values of the same amino acid. This facilitates comparison between the extent of diagenesis in the high-temperature kinetic experiments and fossil biominerals.

**Fig. 10:**
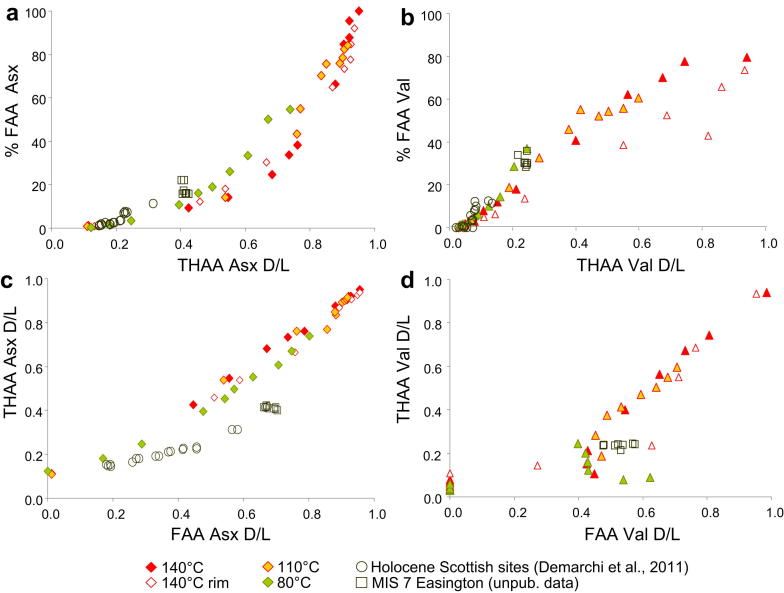
Extent of hydrolysis (% FAA) against the extent of THAA racemisation (*D*/*L*s) for Asx (a) and Val (b) as observed in modern bleached *Patella* heated at 140 °C, 110 °C and 80 °C and in sub-fossil bleached *Patella* of Holocene and Middle Pleistocene age.

Here we compare the extent of Asx and Val diagenesis from the closed-system proteins isolated from *Patella* shells of Holocene (the Scottish sites of Archerfield, Whitegate, Coire and Sand) and Middle Pleistocene ages (Easington raised beach, MIS 7) with the high-temperature data described in this study from both the “bulk” powders sample and the “rim only” powders (Fig. 10). This plot highlights clearly that the patterns of Asx diagenesis in bleached *Patella* shells are different at low and high temperatures. After the initial stages of diagenesis (up to ∼20% FAA in Fig. 10a and FAA *D*/*L* ∼0.2 in Fig. 10c), the fossil samples deviate from the trajectories of the 140 °C and 110 °C experiments (see also Fig. A in Supplementary Information 4, showing that the fossil samples also follow a different trajectory from modern unbleached *Patella* powders, heated at 140 °C). They appear to follow a similar trajectory to the 80 °C data with regards to the release of FAA (Fig. 10a), but the trajectory for Asx racemisation is strikingly different at high and low temperature (Fig. 10c). Therefore, high-temperature experiments do not necessarily mimic Asx diagenesis at low temperatures (see also Goodfriend and Meyer, 1991). Conversely, similar patterns of Val diagenesis can be observed across the different temperatures with regards to extent of hydrolysis versus THAA racemisation (Fig. 10b). The plot of Val FAA *D*/*L* versus THAA *D*/*L* (Fig. 10d) is less informative for Holocene samples due to the low concentrations of FAA Val recovered, which are reflected in variable *D*/*L* values. The Easington specimens fall at lower THAA *D*/*L* values than the high-temperature data for comparable FAA *D*/*L* values; however, the same plot shows that for samples degraded at lower temperatures (i.e. samples heated for 720–960 h for the 80 °C experiment), FAA *DL*s are as high as 0.6, and they subsequently decrease with increasing heating time. This is likely to be due to low FAA Val concentrations for these samples, resulting in higher variability of the *D*/*L* values, but it may also reflect a slow initial release of highly-racemised terminal Val in the FAA pool, followed by increase in the extent of hydrolysis and slowing of the apparent FAA racemisation rate.

Fig. 10 also highlights that different patterns of diagenesis are followed by the proteins isolated from the “bulk” and the “rim only” samples: the % FAA Val, for example, is lower in the rim only than in the bulk sample. This pattern was consistent for all amino acids, although the difference is particularly marked for Val, Ile and Gly. The rim only was targeted for the fossil samples analysed here; however, the pattern of diagenesis followed by these specimens is generally different from both “bulk” and “rim only” high-temperature data.

#### 3.4.2 Extent of diagenesis within Holocene samples

Reaction rates for racemisation and hydrolysis can be estimated for the samples from four dated Holocene sites. Here we focus on Asx and Val as they are two of the most commonly used amino acids in geochronology and because they display different behaviour upon diagenesis (Fig. 10). Reaction rates were calculated using two different approaches:

a) by assuming that the reactions conform to pFOK for these relatively young samples. For Asx we estimated a minimum and maximum rate, due to the scatter of the data and the error associated with the age estimates (Table 8);
b) by applying our model-free approach and estimating the reaction rates relative to the high-temperature data (at 110 °C) (Table 9 and Supplementary Information 1 and 2).

A burial temperature of 10 °C was estimated for the Scottish samples by using a thermal-age model, which accounts for vegetation cover, soil type, depth of burial, as well as geographical location (www.thermal-age.eu, currently under development; arbitrary parameters chosen for the model: depth = 3 m, thermal diffusivity = 0.1 mm^2^ s^−1^).

**Fig. 11:**
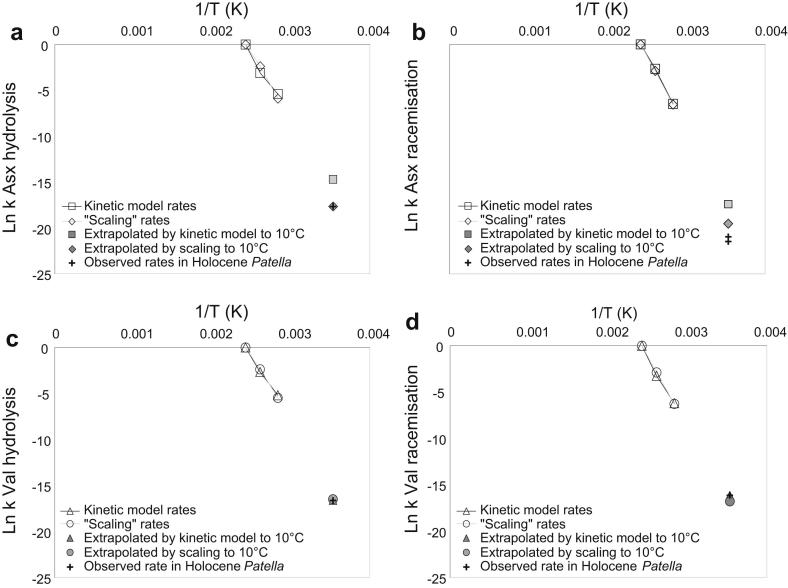
Arrhenius plots for Asx racemisation (a) and hydrolysis (b) and Val racemisation (c) and hydrolysis (d), showing the reaction rates estimated at high temperature with both first-order kinetic models and the scaling method, the extrapolation of the rates at 10 °C and the observed rates in bleached Holocene *Patella* specimens of known age. To ease comparison, all values were adjusted so that the rates for the 140 °C cross the abscises at Ln(*k*) = 0. Racemisation *E*_*a*_ values for the “kinetic model” series were obtained by using a modified first-order rate equation (Eq. (3)) raised to the exponent yielding the best fit to the experimental data. For the extrapolation of Val the “early diagenesis” value (*E*_*a*_ = 104.15 kJ/mol) was used.

On an Arrhenius plot, we then represented (1) the reaction rates at high temperature, obtained by both mathematical models and by “scaling”; (2) the “scaled” reaction rates at 10 °C; (3) the reaction rates extrapolated at 10 °C using the kinetic parameters calculated by applying pFOK rate equations; (4) the observed reaction rates at 10 °C calculated as in point a) (Fig. 11). This allows the accuracy of the extrapolations to be evaluated: if the predicted rate falls close to the observed rate for the Holocene samples, the high temperature experiments (and the model used for the calculation of the kinetic parameters) are able to mimic diagenesis at burial temperatures.

We found that both the pFOK models and the “scaling” approach overestimate the observed racemisation rates for Asx (Fig. 11a) and that the kinetic models overestimate the rate of Asx hydrolysis, whilst the rate obtained by scaling is closer to the observed rate at 10 °C (Fig. 11b). Conversely, rates of both racemisation (Fig. 11c) and hydrolysis (Fig. 11d) of Val appear to be accurately mimicked by high-temperature experiments.

**Fig. 12:**
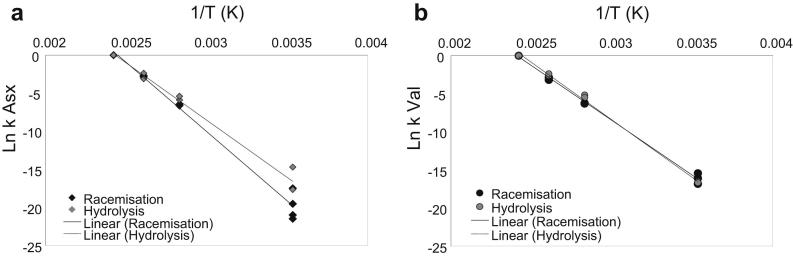
Comparison between the temperature sensitivities of racemisation and hydrolysis at high and low temperature, for Asx (a) and Val (b). To ease comparison, all values were adjusted so that the rates for the 140 °C cross the abscises at Ln(*k*) = 0.

When the temperature sensitivity of hydrolysis and racemisation for both amino acids are compared directly (Fig. 12), it becomes apparent that the patterns highlighted in Fig. 10 can be explained in terms of different *relative* speeds of hydrolysis and racemisation at low and high temperature. At low temperatures (between 10 and 80 °C), hydrolysis of Asx is *relatively* faster than racemisation; therefore for a given THAA Asx *D*/*L* value (e.g. *D*/*L* = 0.8 as in Fig. 10a), the % FAA Asx will be higher at 80 °C (and 10 °C) than at 140 °C. Hydrolysis is likely to expose Asx at the peptide chain termini, slowing the relative rate of racemisation. Conversely at high temperatures, Asx hydrolysis is *relatively* slower than at low temperatures and therefore more in-chain racemisation can occur via a succinimidyl intermediate, accelerating the apparent rate of Asx racemisation. Therefore for any given THAA Asx *D*/*L* (e.g. *D*/*L* = 0.8, as in Fig. 10a), a similar % FAA value is detected in samples heated at 110 °C and 140 °C.

A different situation is found in the case of Val, because the relative speed of hydrolysis and racemisation are similar across different temperatures (Fig. 12b). Therefore, for a given Val THAA *D*/*L*, the % FAA Val will be comparable to a fossil sample, heated at 10 °C, and a kinetic sample, heated at 140 °C.

In conclusion, both % FAA versus *D*/*L* plots and the extrapolations of the reaction rates for hydrolysis and racemisation show that, for Asx, patterns of diagenesis in the intra-crystalline fraction at high temperature do not mimic diagenesis at low burial temperatures. Although this has long been a concern for the AAR geochronology community (Collins and Riley, 2000; Miller et al., 2000; Kaufman, 2006), such a dramatic difference has never been reported in biominerals exhibiting closed system behaviour, for example avian eggshell (Miller et al., 2000); indeed, the study by Miller et al. (2000) examined the extent of epimerisation for both FAA and THAA Ile in fossil and heated modern *Dromaius* eggshell and showed no detectable difference related to temperature between the two datasets. Although their comparison was limited to the *A*/*I* values, not % FAA Ile values, those results indicate that Ile epimerisation displays similar temperature sensitivity to Ile hydrolysis in *Dromaius* eggshell.

However, our study on bleached *Patella* and recent work carried out on the closed-system (bleached powders) proteins isolated from ostrich eggshell (Crisp et al., 2013), *Porites* corals (Tomiak et al., 2013) and other marine molluscs (NEaar laboratory, unpublished data) has highlighted that this divergence between low- and high-temperature data is a phenomenon that can be observed across a range of biominerals.

We suspect this is an inherent characteristic of protein diagenesis in bleached biogenic carbonate and it cannot be overcome by using mathematical models to extrapolate reaction rates at low temperature. We stress that whilst our model-free approach generally is useful for *estimating* rates at burial temperature (see also Tomiak et al., 2013), it cannot be used to *predict* low-temperature rates of reaction. Therefore, it is important that the validity of high-temperature experiments over a range of temperatures be tested by checking their ability to mimic natural diagenesis; for example plots of % FAA versus *D*/*L*, of the extent of racemisation in both the THAA and FAA fractions, as well as the extent of decomposition, are all valuable indicators that can be used simultaneously to check the patterns of protein breakdown within the closed system. A number of studies (e.g. Goodfriend and Meyer, 1991; Goodfriend et al., 1996; Hearty and Kaufman, 2009) have also emphasised the importance of using Holocene shells to constrain the rates at ambient temperatures.

It is likely that performing kinetic experiments at lower temperatures, or using natural samples whose ages and burial temperatures are relatively well known, will be key to obtaining more accurate estimates of the diagenesis rates: the experiment at 80 °C appears to accurately mimic the patterns of Val and Asx diagenesis in *Patella* (Fig. 10). However, these low-temperature experiments are more time-consuming due to the exponential relationship between temperature and diagenesis, resulting in correspondingly long experimental times.

## 4 Conclusions

The patterns of protein breakdown within the intra-crystalline fraction of *P. vulgata* are complex, even though these reactions occur whilst the proteins are effectively retained within a closed system. We observed that the apparent rates of hydrolysis, racemisation and decomposition decrease after prolonged heating at high temperatures. The models of Wehmiller (1980) and Mitterer and Kriausakul (1984) showed that an early diagenetic stage, dominated by hydrolysis of the peptide bonds and extensive racemisation of N-terminal and DKP-bound amino acids, is followed by a stage during which the apparent reaction rates decrease as a result of the increase in the relative abundance of slower-racemising FAA. Leaching of the FAA component and slow hydrolysis of a more resistant protein fraction occurs during the latest stages of diagenesis (Wehmiller, 1980). In the intra-crystalline fraction of proteins examined here, hydrolysis slows either because of limited available water, which results in a second-order (rather than a pseudo-first order) reaction, or, most probably, because of a residual “bound” fraction resistant to hydrolysis (Hoering, 1980; Walton, 1998; Miller et al., 2000; Penkman et al., 2008). Future investigations should focus on the chemical characterisation of this residual bound fraction.

In conclusion, although the proteins investigated appear to undergo predictable diagenesis within a closed system, it is not straightforward to derive mathematical expressions able to describe the relationship between time, temperature and the extent of diagenesis of proteins in bleached powders of *P. vulgata*. Nonetheless, we obtained estimates of the kinetic parameters for hydrolysis, racemisation and decomposition for a range of amino acids by applying well-established models as well as a new “model-free” approach that relies on “scaling” experimental data across a range of temperature and extrapolating the relative reaction rates. All these estimates showed that activation energies for hydrolysis are *lower* than racemisation. Consequently at high temperatures (artificial diagenesis), racemisation is more likely to be the controlling reaction, but at increasingly lower temperatures (sub-fossil *Patella*) this is ceded to hydrolysis. Moreover, there was a clear difference between the diagenetic patterns of Asx when undergoing degradation at high and low temperatures. This can be explained in terms of different relative rates of hydrolysis and racemisation at high and low temperatures, which reflects the complexity of the temperature dependence of each reaction. This pattern has also been found in other bleached biocarbonates (Crisp et al., 2013; Tomiak et al., 2013). It is possible that our observations may be limited to bleached carbonates heated in water, because high-temperature studies on unbleached substrates of different biominerals (e.g. Miller et al., 2000; Kaufman, 2006) did not highlight similar discrepancies between low- and high-temperature data. However, we suggest that the patterns we report for bleached carbonates are detected within the intra-crystalline fraction due to its greater potential for preserving degraded proteins and their breakdown products (e.g. FAA) therefore allowing accurate examination of the diagenesis patterns. Overall, although we do not dismiss the usefulness of high-temperature experiments for providing an insight in protein breakdown, we stress the need to carefully evaluate high-temperature data prior to their use in extrapolating kinetic parameters to estimate the diagenesis rates at various burial temperatures.

A potential way forward for temperature-sensitivity studies may be to investigate proteins and peptide fragments of known amino acid sequences. Past studies of synthetic peptides have yielded fundamental information for our understanding of the mechanisms of racemisation as well as bringing to light the limitations of some of the kinetic models commonly used in AAR geochronology. Recent methodological developments in protein mass spectrometry have opened up the possibility of tackling the issue of the mechanisms of diagenesis. A mechanistic understanding of diagenesis is difficult, because of the complex and variable nature of the process. However, it is important that the temperature sensitivity of the pathways involved in the degradation of amino acids commonly used for geochronology, such as Asx, are better clarified, as this would contribute to improve the accuracy of AAR as a geochronometer.

## Figures and Tables

**Table 1:** Heating times and temperatures used for the kinetic experiments performed on the intra-crystalline proteins in *Patella vulgata*.

Temperature	Heating time (hours)										
80 °C (bulk)	0	24	96	480	720	960	1443	2160	3601	5738	
110 °C (bulk)	0	24	120	240	384	480	720	840	960	1200	
140 °C (bulk)	0	1	2	4	6	8	24	48	72	96.75	240
140 °C (rim only)	0	1	2	6	24	48	96	240			

**Table 2:** Hydrolysis rates (*k*, s^−1^) for Asx, Ala, Ser, Val, Ile and Leu obtained by interpreting the release of FAA at high temperature as a first-order irreversible reaction (Eq. (2)) and coefficients of determination (*R*^2^) for the relationship obtained at three temperatures (140 °C, 110 °C and 80 °C); activation energy *E*_*a*_ (kJ/mol), pre-exponential factor A (s^−1^) for the same amino acids and coefficients of determination (*R*^2^) for the Arrhenius relation. E.D. = Early Diagenesis, where values used for the apparent rate of hydrolysis were limited to % FAA <20 for Leu, Ile and Val.

pFOK	*k* 140 °C (s^−1^)	*R*^2^ 140 °C	*k* 110 °C (s^−1^)	*R*^2^ 110 °C	*k* 80 °C (s^−1^)	*R*^2^ 80 °C	*E*_*a*_ (kJ/mol)	*A* (s^−1^)	*R*^2^
Asx	-9E-06	0.99	-4E-07	0.87	-4E-08	0.95	108	3E+08	0.98
Ser^a^	-1E-05	0.97	-1E-06	0.95	-8E-08	0.95	98	2E+04	0.99
Ala^a^	-1E-05	0.96	-8E-07	0.81	-7E-08	0.94	100	4E+07	0.99
Val	-2E-06	0.73	-2E-07	0.83	-2E-08	0.98	93	1E+06	0.99
Val E.D.	-7E-06	0.98	-5E-07	0.99	-2E-08	0.94	118	7E+09	1.00
Leu	-2E-06	0.77	-2E-07	0.75	-3E-08	0.96	84	8E+04	0.99
Leu E.D.	-9E-06	0.97	-6E-07	0.98	-3E-08	0.99	115	3E+09	0.99
Ile	-2E-06	0.82	-2E-07	0.88	-2E-08	0.92	93	1E+06	0.99
Ile E.D.	-4E-07	0.99	-6E-06	0.94	-9E-09	0.98	132	3E+11	0.99

a Ala and Ser values included only up to ∼75% FAA Ala and 78% FAA Ser.

**Table 3:** Rates of release of free amino acids estimated with the scaling method: effective activation energy *E*_*a*_ (kJ/mol), % FAA range considered for the fitting of the third-order polynomial functions to the 110 °C data, reaction rates relative to the 110 °C data (i.e. scaling factor), sum of least squares for the fitting.

Scaling	*E*_*a*_ (kJ/mol)	Scaling of the 80 °C data to the 110 °C data			Scaling of the 140 °C data to the 110 °C data		
Relative rate	Range of fitting (% FAA)	Sum of least squares	Relative rate	Range of fitting (% FAA)	Sum of least squares
Asx	115	0.0427	10–46	0.01	12.3	12–45	0.01
Asx^a^	115	0.0417	12–45	0.01	12.3	12–45	0.01
Glx	119	0.0375	5–16	0.00	13.7	5–20	0.00
Glx^a^	120	0.0399	6–13	0.00	13.0	6–13	0.00
Ser	87	0.1550	9–17	0.02	11.8	9–2	0.01
Ser^a^	98	0.1098	10–13	0.00	14.7	10–13	0.01
Gly	128	0.0406	12–47	0.01	23.6	14–69	0.11
Gly^a^	123	0.0397	15–47	0.01	17.4	15–47	0.00
Ala	111	0.0730	11–83	0.14	17.8	8–85	0.01
Ala^a^	111	0.0730	11–83	0.14	17.8	11–83	0.01
Val	118	0.0452	2–39	0.01	15.6	4–63	0.00
Val^a^	119	0.0451	4–39	0.01	15.9	4–39	0.00
Phe	119	0.0371	6–31	0.00	13.3	2–50	0.00
Phe^a^	120	0.0371	6–31	0.00	14.1	6–31	0.00
Leu	118	0.0383	7–44	0.03	13.0	8–62	0.00
Leu^a^	118	0.0379	8–44	0.03	13.2	8–44	0.00
Ile	121	0.0384	3–31	0.01	15.5	2–50	0.01
Ile^a^	119	0.0384	3–31	0.01	13.8	3–32	0.00

a Values were obtained by truncating the data series at the same % FAA.

**Table 4:** Racemisation rate constants (2 k, s^−1^) for THAA Asx, Ala, Ser, Val, Ile and Leu obtained by applying Eq. (3); exponent *n* used to transform the first-order rate equation; coefficients of determination (*R*^2^) for the linear regression at each temperature; kinetic parameters (*E*_*a*_ and A) and coefficients of determination (*R*^2^) for the Arrhenius relation.

CPK	*n*	2 k 140 °C (s^−1^)	*R*^2^ 140 °C	2 k 110 °C (s^−1^)	*R*^2^ 110 °C	2 k 80 °C (s^−1^)	*R*^2^ 80 °C	*E*_*a*_ (kJ/mol)	*A* (s^−1^)	*R*^2^
Asx	1.2	9E-05	0.91	1E-05	0.98	4E-07	0.98	110	4E+09	0.99
Asx	1.9	1E-03	0.98	9E-05	0.99	2E-06	0.99	131	3E+13	0.99
Glx	1.2	9E-05	0.98	2E-06	0.97	8E-08	0.91	141	3E+13	0.99
Glx	1.3	1E-04	0.97	2E-06	0.98	9E-08	0.92	141	3E+13	0.99
Ser^a^	1.2	6E-04	0.96	4E-05	0.79	4E-07	0.88	135	3E+13	0.96
Ser^a^	2.8	2E-02	0.97	3E-04	0.96	9E-06	0.93	149	5E+16	0.99
Ala^b^	1.2	1E-04	0.89	9E-06	0.95	2E-07	0.99	126	5E+11	0.99
Ala^b^	1.7	6E-04	0.95	4E-05	0.91	6E-07	0.99	140	2E+14	0.99
Val	1.2	7E-05	0.9	9E-07	0.99	4E-08	0.97	150	2E+14	0.98
Val	0.5	5E-06	0.99	2E-07	0.97	1E-08	0.96	125	2E+10	0.99
Leu	1.2	1E-04	0.91	4E-06	0.92	7E-08	0.99	147	2E+14	0.99
Leu	0.4	8E-06	0.99	4E-07	0.99	2E-08	0.98	121	7E+09	0.99
Ile	1.2	3E-05	0.98	9E-07	0.99	4E-08	0.99	133	7E+11	0.99
Ile	1	2E-05	0.99	6E-07	0.98	3E-08	0.98	131	2E+11	0.99

a Ser values included only up to Ser THAA *D*/*L* = 0.91 (140 °C experiment) and Ser THAA *D*/*L* = 0.86 (110 °C experiment) and excluded the 120 h time point for the 110 °C experiment (outlier).

b Excluding the 840 h time point for the 110 °C experiment (outlier).

**Table 5:** Relative rates of racemisation estimated with the scaling method: effective activation energy *E*_*a*_ (kJ/mol), THAA *D*/*L* range considered for the fitting of the of the third order polynomial function to the 110 °C data, reaction rates relative to the 110 °C data (i.e. scaling factor), sum of least squares for the fitting.

Scaling	*E*_*a*_ (kJ/mol)	Scaling of the 80 °C data to the 110 °C data			Scaling of the 140 °C data to the 110 °C data		
Relative rate	Range of fitting (THAA *D*/*L*)	Sum of least squares	Relative rate	Range of fitting (THAA *D*/*L*)	Sum of least squares
Asx	137	0.0189	0.43–0.84	0.01	16.0	0.53–0.93	0.01
Asx^a^	133	0.0175	0.43–0.85	0.00	12.5	0.43–0.85	0.01
Glx	145	0.0181	0.22–0.45	0.00	23.3	0.23–0.79	0.02
Glx^a^	140	0.0186	0.24–0.46	0.00	18.9	0.24–0.46	0.01
Ser	133	0.0084	0.31–0.84	0.05	12.6	0.52–0.90	0.08
Ser^a^	141	0.0086	0.51–0.84	0.07	10.8	0.50–0.85	0.03
Ala	128	0.0318	0.25–0.64	0.00	14.6	0.26–1.00	0.13
Ala^a^	128	0.0318	0.25–0.64	0.00	17.4	0.25–0.64	0.01
Val	128	0.0353	0.06–0.27	0.00	19.4	0.06–0.57	0.03
Val^a^	126	0.0415	0.06–0.26	0.00	20.5	0.06–0.26	0.01
Phe	137	0.0217	0.13–0.41	0.02	18.2	0.17–0.81	0.20
Phe^a^	139	0.0254	0.15–0.40	0.01	22.8	0.15–0.40	0.05
Leu	128	0.0377	0.09–0.45	0.01	17.5	0.13–0.92	0.06
Leu^a^	131	0.0346	0.13–0.46	0.00	22.5	0.13–0.46	0.01
Ile	133	0.0284	0.06–0.30	0.00	20.5	0.11–0.73	0.04
Ile^a^	134	0.0326	0.10–0.29	0.00	24.5	0.10–0.29	0.00

a Values were obtained by truncating the data series at the same *D*/*L*.

**Table 6:** Apparent decomposition rates (*k*, s^−1^) for Asx and Ser obtained by applying Eq. (4); coefficients of determination (*R*^2^) for the linear regression at each temperature; kinetic parameters (*E*_*a*_ and *A*) and coefficients of determination (*R*^2^) for the Arrhenius relation.

pFOK	*k* 140 °C (s^−1^)	*R*^2^ 140 °C	*k* 110 °C (s^−1^)	*R*^2^ 110 °C	*k* 80 °C (s^−1^)	*R*^2^ 80 °C	*E*_*a*_ (kJ/mol)	*A* (s^−1^)	*R*^2^
Asx	-2E-06	0.93	-1E-07	0.87	-9E-09	0.60	108	9E+07	0.99
Ser	-1E-05	0.91	-7E-07	0.90	-6E-08	0.84	103	1E+08	0.99

**Table 7:** Relative rates of Asx and Ser decomposition estimated with the scaling method: effective activation energy *E*_*a*_ (kJ/mol), [THAA]_aa_/[Total THAA]_0_ (as defined in Eq. (4)) range considered for the fitting of the third order polynomial function to the raw 110 °C data, reaction rates relative to the 110 °C data (i.e. scaling factor), sum of least squares for the fitting.

Scaling	*E*_*a*_ (kJ/mol)	Scaling of the 80 °C data to the 110 °C data			Scaling of the 140 °C data to the 110 °C data		
Relative rate	Range of fitting	Sum of least squares	Relative rate	Range of fitting	Sum of least squares
Asx	146	0.0077	0.27–0.25	0.00	10.0	0.26–0.2	0.00
Ser	131	0.0250	0.07–0.04	0.00	16.1	0.07–0.00	0.00
[Ser]/[Ala]	131	0.0246	0.86–0.22	0.01	15.8	0.66–0.00	0.02

**Table 8:** Apparent rates of hydrolysis and racemisation for Asx and Val estimated in *Patella* specimens from Scottish Holocene sites of known age assuming a first-order rate model.

pFOK	Reaction	*k* (s^−1^)	*R*^2^
Asx	Hydrolysis (min)	-2E-13	0.90
Asx	Hydrolysis (max)	-4E-13	0.99
Asx	Racemisation (min)	3E-13	0.76
Asx	Racemisation (max)	5E-13	0.92
Val	Hydrolysis	-4E-13	0.91
Val	Racemisation	2.5E-13	0.69

**Table 9:** Relative rates of hydrolysis and racemisation for Asx and Val estimated in *Patella* specimens from Scottish Holocene sites of known age by the “scaling” approach. Effective activation energies estimated over the full temperature range, i.e. between 10 and 140 °C.

Scaling	*E*_*a*_ (kJ/mol)	Scaling of the 10 °C data to the 110 °C data		
Relative rate	Range of fitting	Sum of least squares
Asx hydrolysis	133	2.79E-07	% FAA: 2–15	0.001
Val hydrolysis	125	9.18E-07	% FAA: 0–10	0.001
Asx racemisation	152	3.28E-08	THAA *D*/*L*: 0.18–0.34	0.003
Val racemisation	130	5.51E-07	THAA *D*/*L*: 0.04–0.12	0.000
